# Algorithms used in medical image segmentation for 3D printing and how to understand and quantify their performance

**DOI:** 10.1186/s41205-022-00145-9

**Published:** 2022-06-24

**Authors:** Magdalene Fogarasi, James C. Coburn, Beth Ripley

**Affiliations:** 1 US Food and Drug Administration, Center for Device and Radiological Health, Silver Spring, MD 20993 USA; 2 US Food and Drug Administration, Office of the Chief Scientist, Silver Spring, MD 20993 USA; 3 Fischell Department of Bioengineering, University of Maryland, College Park, MD 20742 USA; 4 US Department of Veterans Affairs, Veterans Health Administration, Office of Healthcare Innovation and Learning, Seattle, WA 98109 USA

**Keywords:** Patient-specific anatomical model, 3D printing, Geometric variation, Medical image segmentation, Segmentation variation, Algorithms, Hospital, Point of care

## Abstract

**Background:**

3D printing (3DP) has enabled medical professionals to create patient-specific medical devices to assist in surgical planning. Anatomical models can be generated from patient scans using a wide array of software, but there are limited studies on the geometric variance that is introduced during the digital conversion of images to models. The final accuracy of the 3D printed model is a function of manufacturing hardware quality control and the variability introduced during the multiple digital steps that convert patient scans to a printable format. This study provides a brief summary of common algorithms used for segmentation and refinement. Parameters for each that can introduce geometric variability are also identified. Several metrics for measuring variability between models and validating processes are explored and assessed.

**Methods:**

Using a clinical maxillofacial CT scan of a patient with a tumor of the mandible, four segmentation and refinement workflows were processed using four software packages. Differences in segmentation were calculated using several techniques including volumetric, surface, linear, global, and local measurements.

**Results:**

Visual inspection of print-ready models showed distinct differences in the thickness of the medial wall of the mandible adjacent to the tumor. Volumetric intersections and heatmaps provided useful local metrics of mismatch or variance between models made by different workflows. They also allowed calculations of aggregate percentage agreement and disagreement which provided a global benchmark metric. For the relevant regions of interest (ROIs), statistically significant differences were found in the volume and surface area comparisons for the final mandible and tumor models, as well as between measurements of the nerve central path. As with all clinical use cases, statistically significant results must be weighed against the clinical significance of any deviations found.

**Conclusions:**

Statistically significant geometric variations from differences in segmentation and refinement algorithms can be introduced into patient-specific models. No single metric was able to capture the true accuracy of the final models. However, a combination of global and local measurements provided an understanding of important geometric variations. The clinical implications of each geometric variation is different for each anatomical location and should be evaluated on a case-by-case basis by clinicians familiar with the process. Understanding the basic segmentation and refinement functions of software is essential for sites to create a baseline from which to evaluate their standard workflows, user training, and inter-user variability when using patient-specific models for clinical interventions or decisions.

## Introduction

Many clinicians are beginning to use 3D printing (3DP), a form of additive manufacturing (AM), to make anatomic models for surgical planning, patient education, and more [1–3]. Over the last decade, traditional manufacturers have used 3DP to fabricate patient-specific medical devices [4] and the breadth of medical image segmentation algorithms has evolved extensively [5–7]. However, the more recent trend is for health care systems to bring 3D printing capabilities within the walls of the hospital at the point of care. With increased implementation at the point of care, health care facilities need to develop methods that ensure these devices are safe and do not increase risk to the patient. In December 2021, the US Food and Drug Administration (FDA) released a discussion paper on the types of 3DP activities projected to be undertaken at the point of care and how they might be regulated [8, 9]. Common use cases include patient-specific implants, surgical cutting guides, and anatomic models [10–13]. Anatomic models may improve surgical outcomes and provide tactile stimulus during surgical planning [14, 15], are the digital base of patient-specific surgical guides, and are the most widespread use case of 3DP in healthcare facilities. Anatomical models may be perceived to have a lower risk compared to cutting guides and implants, but they are still considered medical devices [16] and point of care manufacturers must ensure that these models meet clinical requirements for safety and efficacy. This takes experience and a systematic approach developed from understanding critical attributes of the digital design and fabrication process.

**Fig. 1:**
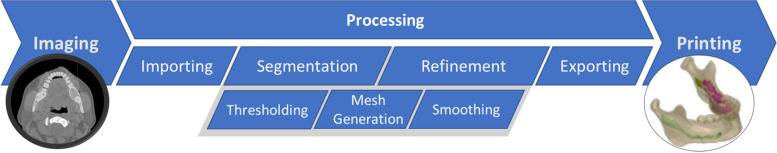
Basic workflow for processing patient image volumes into 3D printable models

Converting volumetric medical imaging data sets into a 3D printable format involves many steps: a digital pipeline (Fig. 1), where software algorithms are used to isolate anatomic regions of interest (ROI) (referred to as segmentation), converting the ROI volume to a surface mesh, and cleaning up edges or errors (referred to as smoothing) [17].Many algorithms and methods have been developed to help users identify boundaries between complex anatomic regions in medical images by automating segmentation and then increasing accuracy through refining processes. These processes can have clinically relevant effects on the final product, depending on the ROI’s important features and how the algorithms handle them. Knowledge of key algorithms and parameters can help a user to determine how the software itself will affect the accuracy of the anatomic model and, ultimately, patient safety, irrespective of user variability or other factors.

Many studies have identified absolute differences in accuracy between programs for specific software, [15, 18], described 3D printing workflows for different applications [19–21], and discussed printing best practices [22, 23]. In this paper, the behaviors of different mathematical algorithms used in ROI segmentation and refinement are reviewed to highlight key features that can affect the transformation of medical images into 3D printable models. Next, these concepts are synthesized and illustrated with a real-world clinical case segmented using four different software packages that use various algorithms for segmentation and refinement, including FDA cleared and noncleared software that are either proprietary or open-source. Finally, several metrics are used to quantify local and global geometric variation in 3D models introduced from software workflows. This information provides a framework and tool to assist engineers and clinicians in evaluating and implementing their own processes. The comparison metrics used here can be extended and repeated with other software programs and workflows not described here.

### Background: image acquisition and representation of digital anatomy for 3D printing

Volumetric clinical imaging data sets (e.g. computed tomography (CT) and magnetic resonance imaging (MRI)) are typically the source data for 3DP models. These data sets are comprised of volumetric pixels, more commonly referred to as voxels. Slice thickness (z)and pixel size (xy) are a function of scanner hardware parameters, assigned field of view, and matrix size; they affect the overall size and uniformity (isotropy) of voxels. For the same exposure, larger voxels have a higher signal to noise ratio, but at the cost of decreased spatial resolution and increased volume-averaging from adjacent structures co-located within a given voxel [24–28]. Imaging protocols optimized for creation of patient specific 3D models must weigh these trade-offs, and additionally consider contrast requirements, timing, and overall patient positioning; these 3D printing optimized protocols may be different than standard clinical imaging protocols used for diagnosis [4, 29, 30]. Segmentation is the act of assigning voxels that contain an anatomic region of interest (ROI) to a resulting volume or mask. This stair-step appearance is the result of the voxel size and shape and does not represent the true organic contours of tissues or structures. Instead, it contains a portion of adjacent tissue contours when they happen to fall within a given voxel. This stair-step appearance is often softened during the creation of 3D printable surface meshes from volumetric data. This is because surface meshes are essentially “wrapping” the ROI in a fitted net composed of triangles which can smooth out the blocky voxels.

**Fig. 2:**
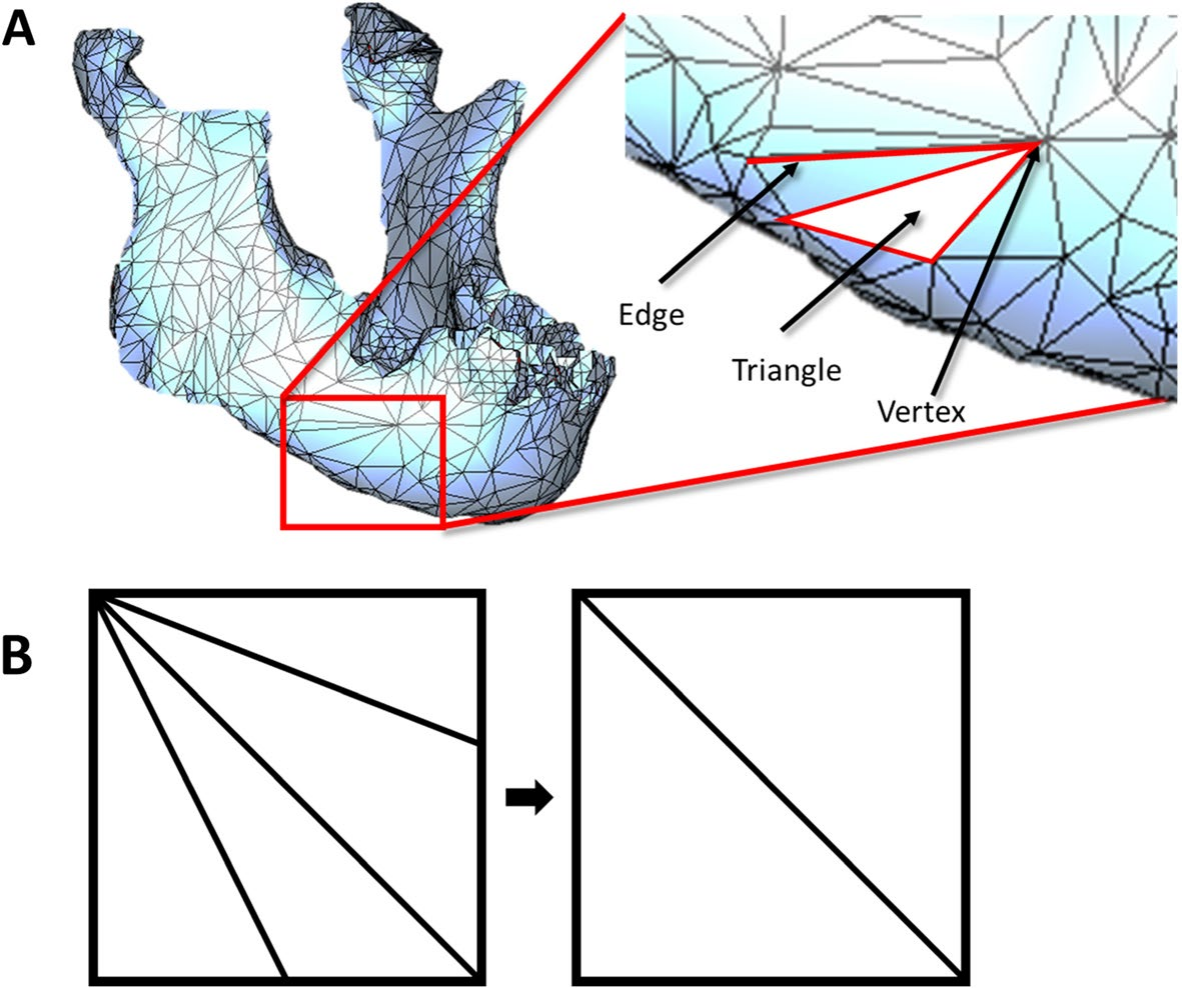
Meshing Basics **A** Solid mesh of a mandible with major mesh features identified. **B** Decimation of features while maintaining fidelity of features

In this analogy, the net consists of intersections or vertices connected by edges. The space between any three vertices is called a face. (Fig. 2A). Most surfaces begin with uniform size triangles. Increasing the number of triangles in a mesh, much like increasing resolution of the scan, will better approximate curved features. However, dense meshes can be computationally burdensome, so a balance must be struck between complexity and dimensional fidelity. Note that reducing the number of triangles does not always mean that the fidelity will be reduced (Fig. 2B).

### Background: Algorithms

Studies of digital workflows found that the largest influences on model accuracy were scan quality and manual segmentation for complex soft tissue cases [17, 26, 30–33]. Recent algorithm improvements for both segmentation and refinement have increased the availability of automated methods [34–36], correspondingly increasing the required user knowledge of algorithms and ability to validate them. The two image processing steps with the greatest chance to impact the final model accuracy are mask editing and mesh smoothing. Once an initial mask is defined, region growing, which examines voxels neighboring a seed region, can then be performed to refine the thresholded mask [37]. Programs can then semi-automatically refine segmentation masks using various methods to locate the ROI contours and create the 3D surface mesh. Two examples of these methods are active contours [38, 39] and region competition [40], which both identify and move a contour towards similar nearby voxels while minimizing the contour’s deformation. Segmentation results in a volumetric ROI, which is then converted into a surface mesh. The newly created meshes can be smoothed and refined - terms that often overlap. *Refinement* may include any of several methods to increase the fidelity of the mask or the mesh in specific areas where feature resolution is needed. *Smoothing* will refer to algorithms that work on the mesh to decrease quick changes in direction of contours and flatten features according to user defined settings. *Decimation* can reduce the number of triangles that make up a mesh - decreasing file size and complexity while ideally preserving topology [41]. Meshes made from patient images tend to be very complex and decimation reduces computational load.

Most mesh smoothing algorithms function by iterating through the mesh and relocating vertices according to mathematical restrictions that optimize the mesh to a user-set parameter. However, different smoothing algorithms give different results. This will typically reduce mesh complexity (increase triangle size) in flat areas and increase complexity in areas with many geometric features.

The most common smoothing algorithms implement Laplacian smoothing [42, 43], a vertex-based technique that iteratively converges a curve toward a point. This typically shrinks the volume of the mesh and pure Laplacian implementations do not correct for mesh shrinkage. Modifications such as Taubin smoothing [44] include a compensating inflation step after each mesh shrinkage step. Similar methods such as angle-based [45], bilateral [46], and curvature [47] have been implemented to optimize smoothing in a manner that preserves details (sharp points, small radius curvatures, or thin walls) in the mesh. Most programs include user-selectable options to preserve small features and boundaries. Methods of optimally smoothing a mesh have been an important topic for a long time and they often work extremely well for regular, known or smooth shapes. New implementations and optimizations are constantly being introduced [48–50] to deal with new cases. A summary of popular smoothing algorithms with visual examples is located in Table 1. It is incumbent on the user to identify the characteristics of their software and determine which are most important for their clinical use cases.

**Table 1:**
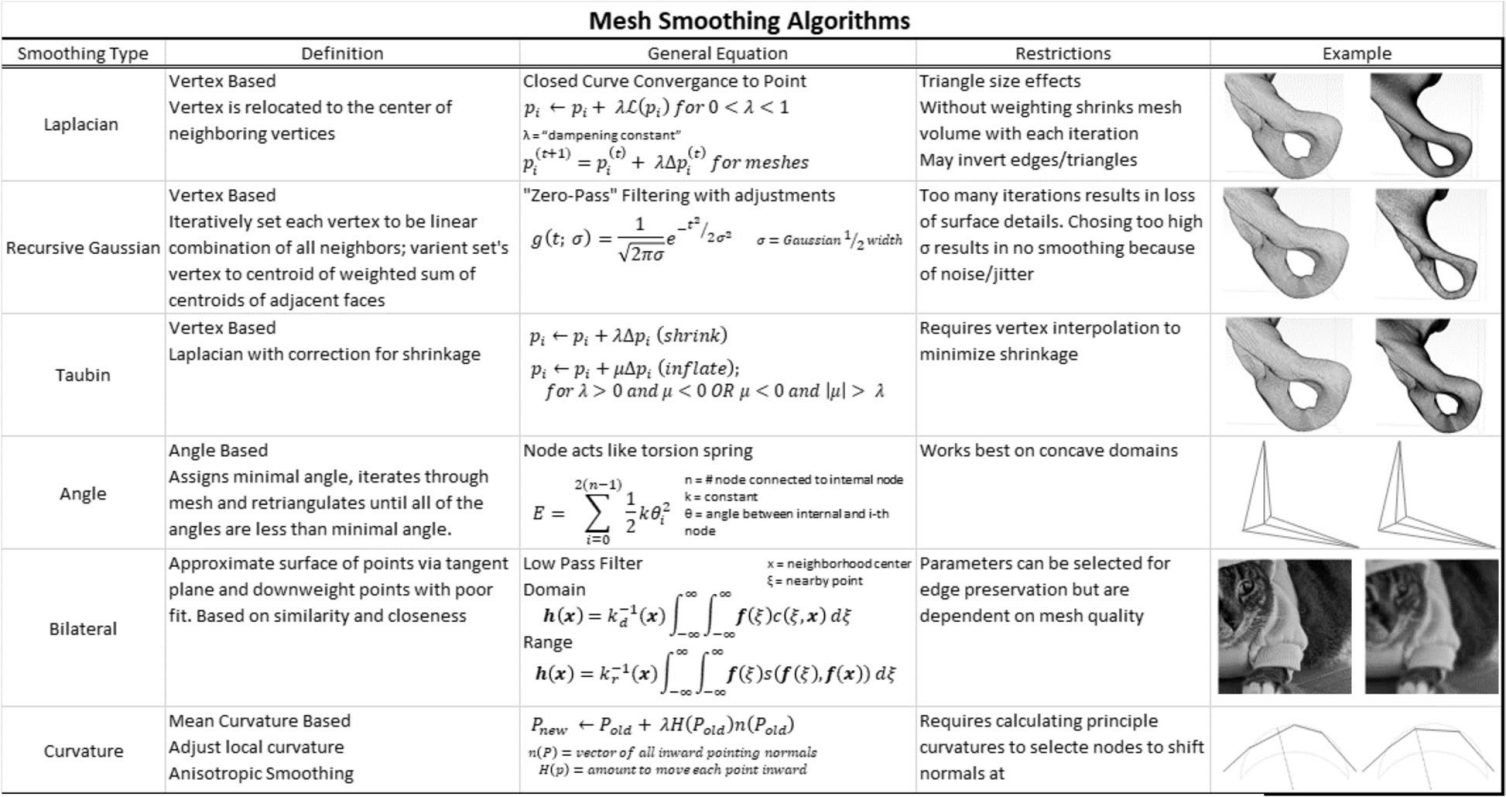
Summary of most common smoothing algorithms used in segmentation software. Images in Example column demonstrate implementation of the algorithm before (left) and after (right) implementation.

## Materials & methods

### Materials

The example data set was a cranio-maxillofacial CT scan used in a 2018 RSNA hands-on 3D printing training module [51] (slice thickness 1.0 mm, no gantry tilt, 512 × 512 FOV, 0.3319 mm pixel spacing, 16-bit, 120 kVP, FC80 kernel, and 40 mA). The completely deidentified dataset, available upon request, was provided by Materialise NV (Leuven, Belgium) and was used after receiving approval from the institutional review board. All methods were carried out in accordance with relevant guidelines and regulations. The dataset features a large right mandibular tumor; the tumor is adjacent to both a nerve and an impacted wisdom tooth. This file was chosen because it contains several ROIs with varying degrees of segmentation complexity to challenge different aspects of the digital pipeline. All input images were in Digital Imaging and Communications in Medicine (DICOM) format, and output meshes were saved as STL files.

The four programs selected for this study were Dicom2Print (3D Systems, South Carolina USA), Mimics (Materialise NV; Leuven, Belgium), 3D Slicer (Brigham and Women’s Hospital, Massachusetts USA), and Simpleware (Synposys; California, USA). Throughout, they will be referred to as Programs 1–4 (not ordered as above) because the goal of the study is not to compare the programs themselves. Rather, the goal is to use the programs as examples of different algorithm implementations and to demonstrate the variation that can occur between the workflows of any segmentation software.

All comparisons were made between the final STL surface meshes output from each program. Full mesh comparisons were made using Magics (23.0, Materialise). Linear dimensions and volumetric measurements were computed in 3-Matic (19.0, Materialise). Statistics were calculated using MATLAB (R2007a, Mathworks).

## Methods

### Digital workflow for STL generation

The process of creating an STL file from DICOM scans follows a similar workflow in all programs, generalized into five main steps: (1) DICOM import, (2) Segmentation, (3) Mesh/Model Generation, (4) Smoothing/Mesh Refinement, and (5) Exporting to STL (or other 3D printer-compatible file format). Initial DICOM imports all maintained the underlying image volume characteristics (e.g., no alterations were made to the source data). As this study was designed to isolate and measure the effects of different algorithms applied throughout the software workflows, a single user (MF) performed all 5 steps of the digital workflow for all conditions in this study (removing any inter-user variability).

The terms automatic, semi-automatic, and manual loosely group interventions by the amount of user intervention required to complete that step of the workflow (Table 2). Regions of interest were segmented in each program, making use of automated and turn-key options when available. For automatic segmentations with default settings, the program-assigned Hounsfield unit range was not adjusted. For semi-automatic thresholding, Hounsfield unit boundaries (minimum and maximum) were set by the user to be the same between programs. Global thresholding based on gray values/Hounsfield units was the principle starting point for bone volume segmentation. Thresholding identified the initial mask or created seed markers starting with bone presets (if available), then editing the mask in later steps. Tumor segmentation was performed by using soft tissue CT presets to generate the initial mask and then editing the mask of the tumor area from the mandible using mask splitting. In some of the programs, it was necessary to manually edit the tumor segmentation layer by layer along the bone tissue interface. Segmentation was performed manually for the nerves in all cases by painting on the mask on each layer visible and using interpolation when available to connect the mask layers. Thresholding was attempted for the nerves but because of their resolution it was not an effective method of segmentation. At this point, meshes were generated (step 3 of 5 in the digital pipeline, Fig. 1). This was automatic for all 4 programs.

**Table 2:**
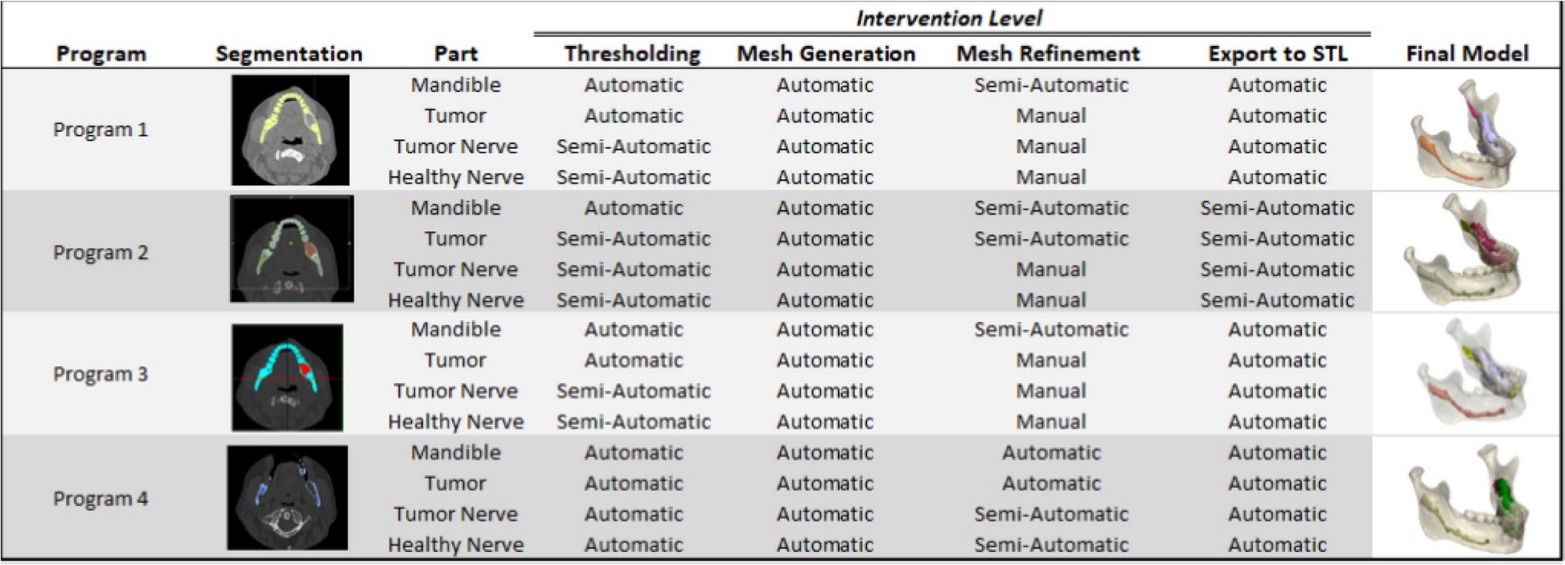
User intervention level at different workflow steps, by program. Automatic designates use of a menu option or built in automatic “turn key” feature for the majority of the segmentation step. Semi-Automatic designates segmentation steps that required user intervention but made use of built-in operation controls where parameters must be set by the user. Manual designates steps that required all or almost all manual user intervention to segment. Final Models after export were used for subsequent calculations.

Mesh refinement (step 4, Fig. 1) used smoothing algorithms that relocated vertices and/or changed the number of triangles (Table 3). Three levels or intensities of smoothing were chosen to best show the changes made by each setting: 0) no smoothing, 1) low smoothing, and 2) high smoothing. Within the semi-automatic intervention level, the amount of user interaction and control varied between programs. In the final step (step 5, Fig. 1), all meshes were exported as STL files, a condition included in the variability measurements.

**Table 3:**
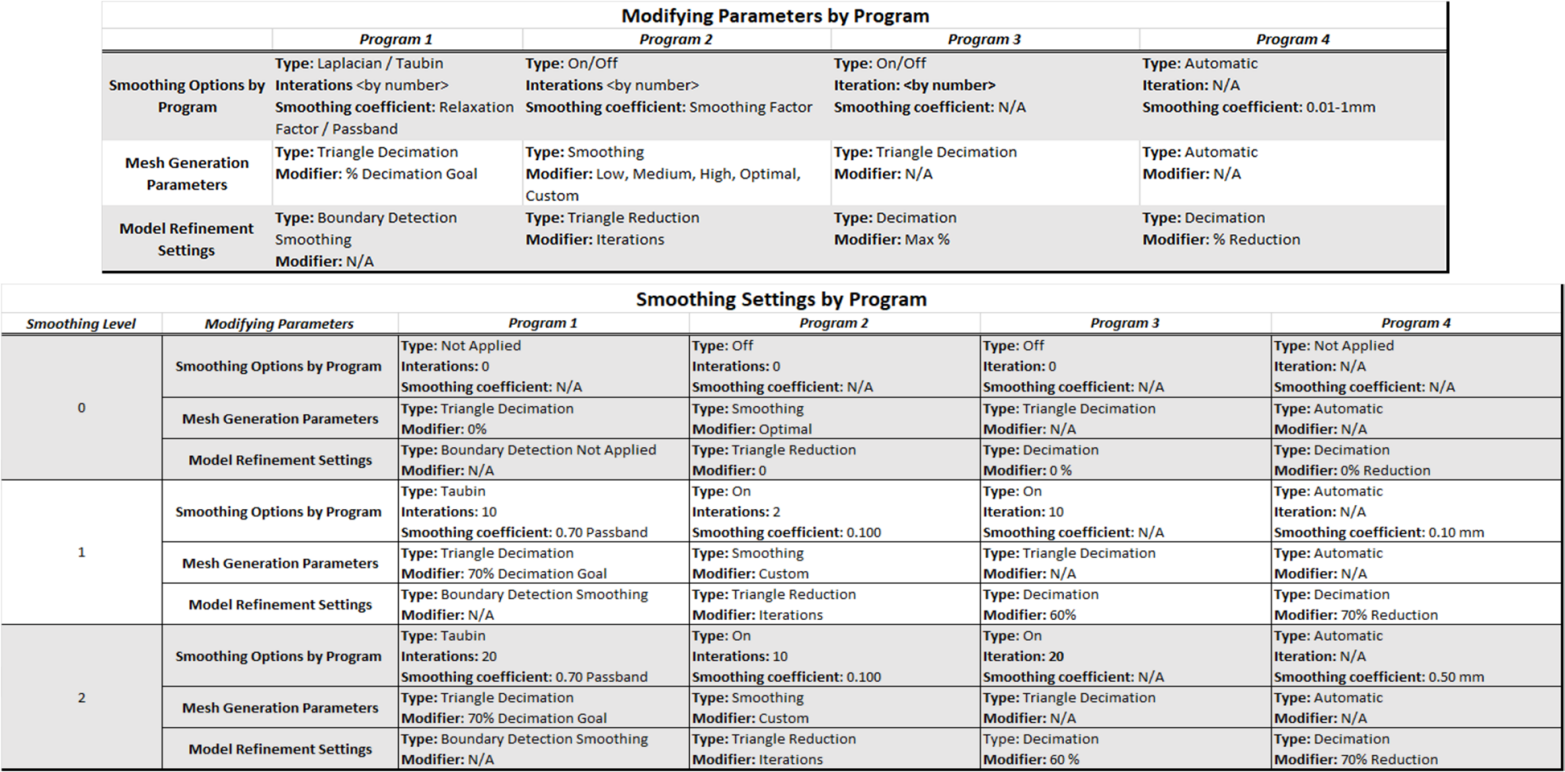
Smoothing settings for each program given the available smoothing options. Groupings were assigned to generate comparable options from disparate software nomenclature and may not represent exact definitions or labels in their respective software. The volume and surface areas of the three models generated at each smoothing level were averaged and used for statistical analysis.

### Comparison of STL files across levels of smoothing and software programs

A total of 12 models were compared, resulting from three smoothing levels (0, 1, 2) performed in each of 4 programs. Measuring the accuracy of anatomic models often presents a conundrum in that ground truth is only available when scanning cadaveric or simulated models that can be measured separately. Given the inability to perform ground truth verification, we evaluated several metrics for their ability to measure the agreement and disagreement between segmented regions of interest. Volume and surface area measurements provide global comparisons between models generated by each software. Linear measurements provide a means for measuring local differences between models.

### Metrics for global comparison

A residual volume comparison available in the literature [52] was used to calculate tumor volumes for all models. These residuals were used to calculate the agreement and disagreement volume and percentages between models (Eqs. 1 and 2), and pairwise statistics were performed. [52, 53]. The agreement (Eq. 1) metric defines the space that is occupied by both models and can be used to assess accuracy and repeatability in models made by different operators or software programs, or smoothing strategies [54]. Disagreement (Eq. 2) defines the space occupied by only one of the models and not the other.

Surface deviation heatmaps were created for all program pairs by calculating the Hausdorff distance, which is a measure of the distance from all points on one surface to the corresponding points on a second surface, to identify all local areas where variation was more prominent.

### Metrics for local comparison

Local comparisons were taken on the final, digital print-ready models output by each program, and allowed for evaluation of software performance on specific ROIs (mandible, tumor, nerve). Linear mandible measurements were performed for all models (across smoothing levels and software programs) with virtual calipers (Magics 23.0) using clinical fiduciary markers [55, 56]. To facilitate linear measurements of the tumor, parallel measurement planes were evenly distributed throughout the tumor region by slicing each STL with identical, parallel datum planes. The centroid of each planar contour was used to measure corresponding X and Y distance to the tumor edges on that contour. Similarly, the alveolar nerve path was measured using the centroid location coordinates of selected coronal slices. Consistent slices were user-selected and evenly distributed along the nerve models.

### Statistics

Comparative analytics between the mandible and tumor STL model surface areas, STL model volumes, and nerve path centroids were calculated using ANOVA (α = 0.05) with Tukey post hoc testing.

## Results

### Overall observations

The time to process the image volume through all segmentation and refinement steps was comparable for most of the programs, although Program 4 took substantially less time. Manual intervention to some degree was required in all cases but the amount of intervention varied by program. User time to segment in order of program was 4.4 h (Program 1), 4.5 h (Program 2), 4.6 h (Program 3), and 2.0 h (Program 4).

**Fig. 3:**
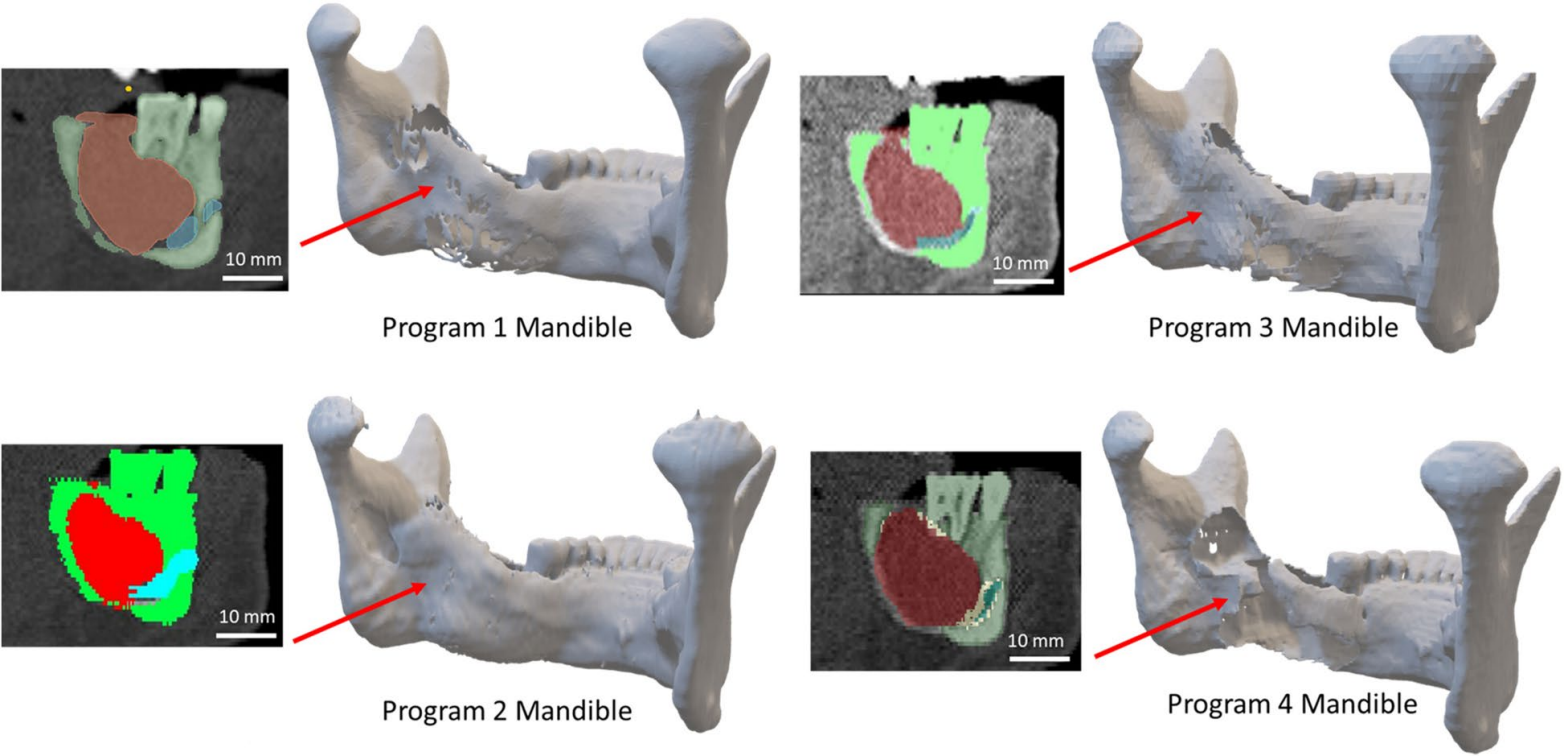
Volume representations of mandible segmentations with accompanying mask previews. Note the differences in segmentation of the interior wall of the mandible and location of the nerve in each cross-section. Models presented are after Smoothing 1 modifications were applied per program

Initial visual inspection of print-ready models showed clear differences in bone contour around the tumor, especially at internal soft tissue boundaries. On the inner left wall of the mandible abutting the tumor, there were noticeable differences in wall thickness, with areas of apparent absent bone as viewed in detail in Fig. 3. Bones with a thin cortical layer are challenging to segment at baseline, and the disruption of the cortex caused by the adjacent tumor compounded the challenge such that most of the programs did not capture all the bone contour along the inner wall.

### Effect of different levels of smoothing on final model STL

We tested 12 conditions, including 3 levels of smoothing within each of the 4 programs. The different smoothing levels created no statistically significant differences in final models within each program, therefore only results of program-to-program comparisons are presented. For linear, surface area, and volume measurements, dimensions were averaged across smoothing levels for each program (values for each program represent *average(Smoothing0, Smoothing1, Smoothing2)*. For heatmap and intersection (agreement/disagreement) calculations, smoothing level 1 models were compared between programs.

### Quantitative differences using global measurements

**Fig. 4:**
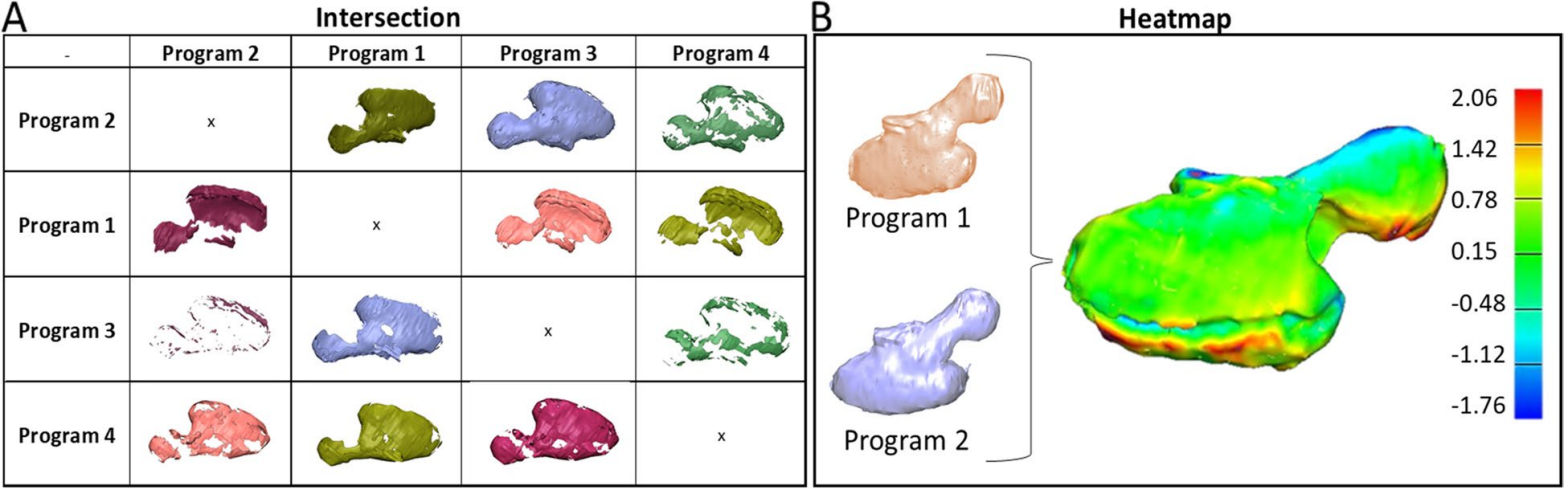
Intersection matrix of tumor models from each program using agreement and disagreement calculations (**A**), Heatmap using Hausdorf distances between two models with largest mismatch, color bar in mm (**B**)

Surface deviation heatmaps and residual volume comparisons were used to assess global inter-model differences for models created using smoothing level 2 in each of the four programs. The pair of models with the worst agreement is presented as it demonstrates the best visualization in a static setting (Fig. 4B). Union and intersection figures (Fig. 4A) highlight variations in the outer contour of the tumor between software programs, locating the areas of greatest mismatch. Agreement and disagreement percentages (Table 4) help to generalize this number, with the disagreement metric providing a better sense of the degree of mismatch.

**Table 4:**
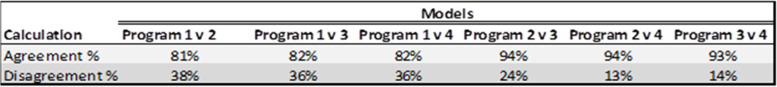
Tumor volume agreement and disagreement, in percent (%), rounded to nearest percent.

Significant differences (*p* < 0.0005) were found for the averaged final models compared between programs, for the following parameters: mandible volumes, tumor volumes, and the mandible surface area (compared separately).

Signed difference heat maps of models from each pair of programs illustrated the most problematic locations and the differences seen between segmentation / refinement processes. The pair of models with the largest differences (Fig. 4B) highlighted the areas of high deviation between programs in red (positive deviation) and dark blue (negative deviation). While deviation magnitudes were less than or equal to 2.06 mm even in the worst case, the location of the differences was along the nerve path. Both of these location-specific metrics draws the user’s attention and scrutiny to regions that were determined to be clinically important before the segmentation process began.

### Local geometric differences

It is not usually possible to find general metrics that capture all the relevant features of a case in one or two numbers. Additional metrics that can show local model variation and features are essential for validating processes and maintaining output quality based on clinically relevant criteria. They are also often easier to compare with the original DICOM data, as linear measurements are easily performed using PACS software.

**Fig. 5:**
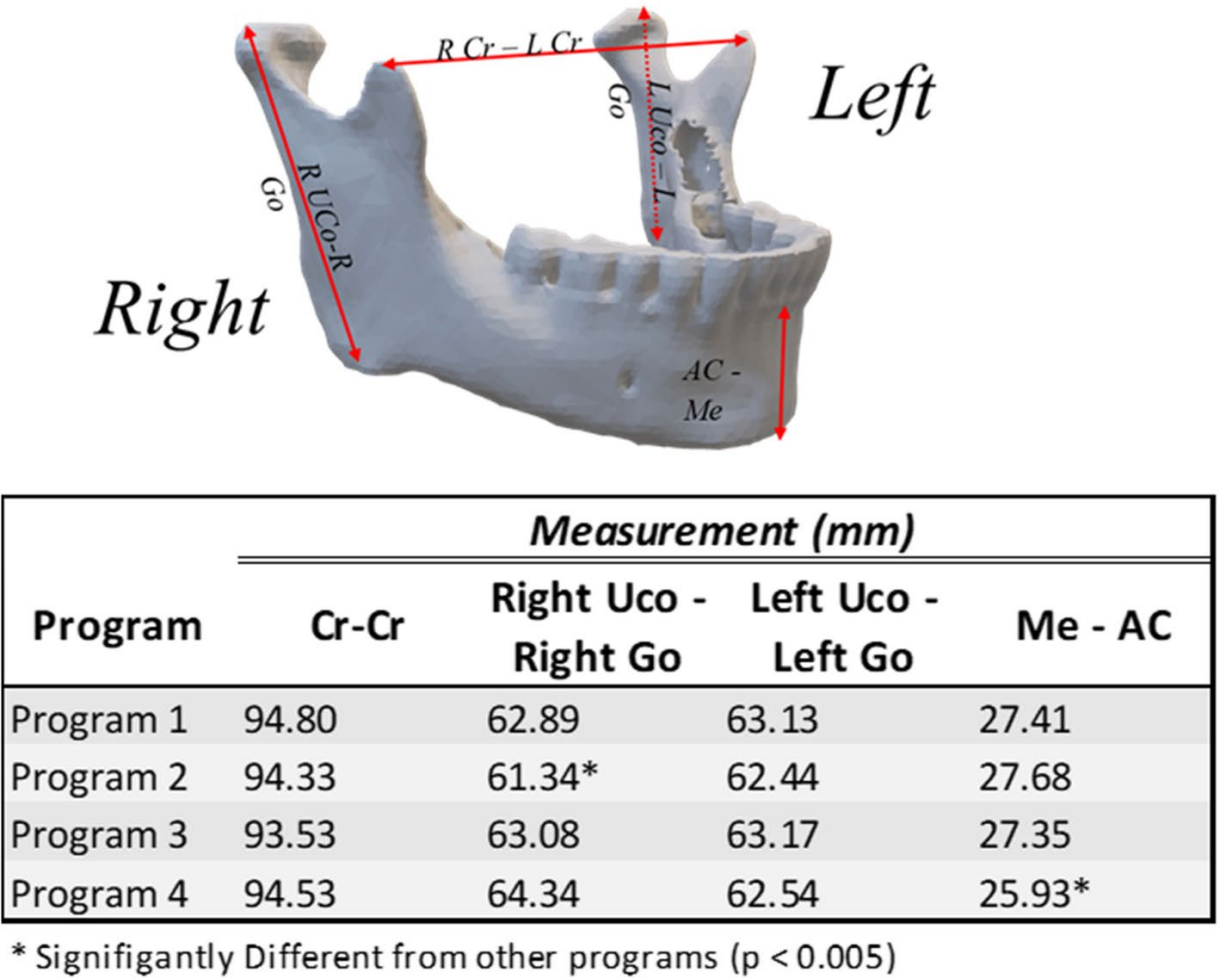
Mandible Linear Measurements. Measurement locations taken (top) and results (bottom). Measurements taken in 3-matic. * ANOVA (α = 0.5). Tukey–Kramer Q4,6,0.05 = 4.065

Two point-to-point length measurements on the mandible differed with statistical significance using ANOVA (α = 0.05) with Tukey post-hoc: Right UCo – Right Go for Program 2 (*p* < 0.005), and Me-AC for Program 4 (*p* < 0.005) differed from the other programs (Fig. 5) but these values did not appear to be clinically significant.

Local measurements were particularly needed in assessing differences in segmentation of the nerve.

**Fig. 6:**
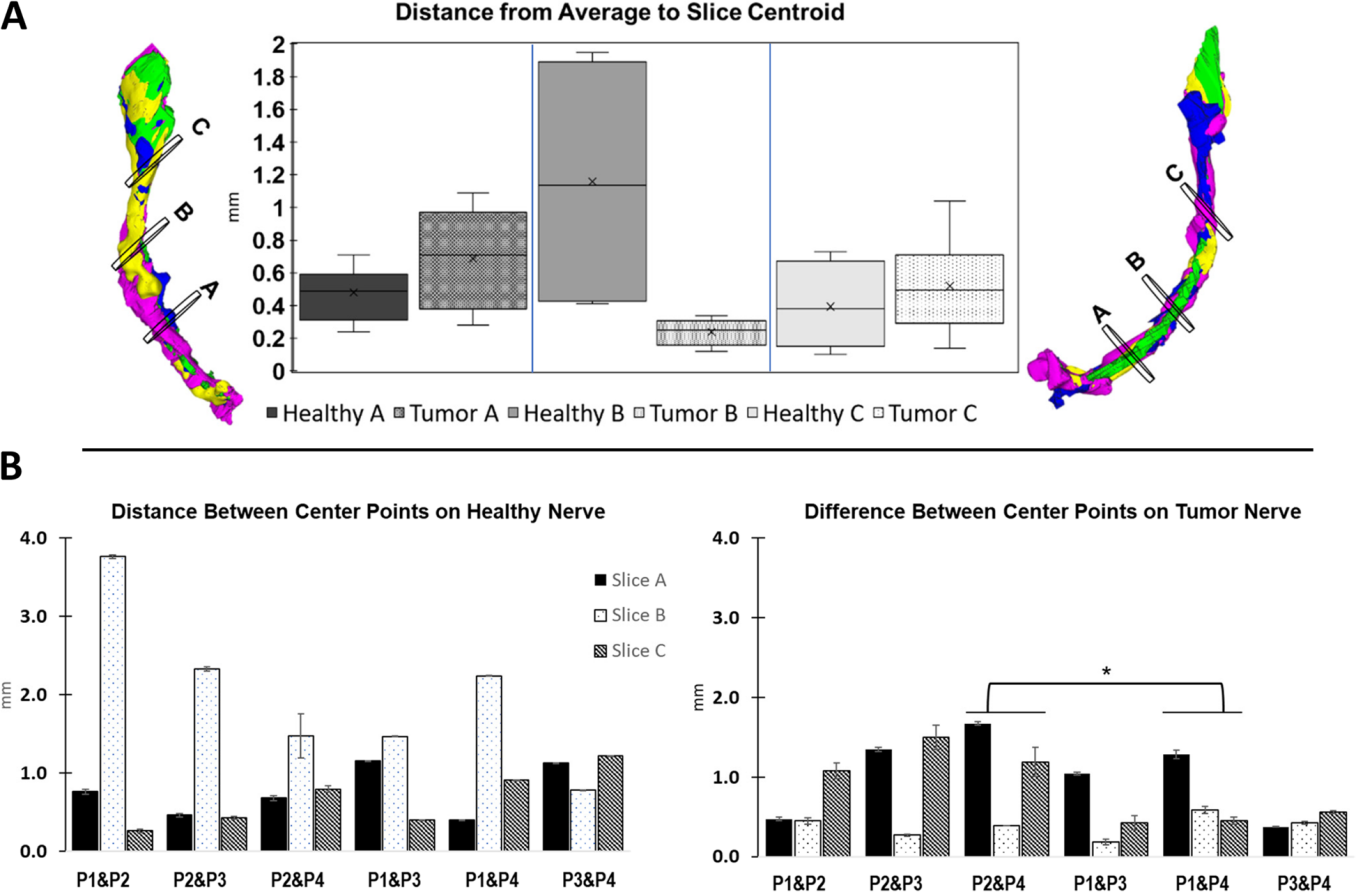
Summary of deviations in geometry between nerve segmentations. **A** Differences between programs (P1-P4) across all slices Healthy Nerve on left, Tumor Nerve on right. (* = *p* < 0.05, Tukey–Kramer, Q6,39,0.05 = 4.237)) **B** Differences between all programs per slice to centroid of each sliced based on the centroids of all models per slice alternating Healthy and Tumor Nerve centroid distance distributions for each slice. STL Stacks of Healthy (L) and Tumor (R) Nerves and location of slices on each

Visual inspection of Intersection metrics and stacked nerve segmented models showed local differences in undulations of the nerve path (Fig. 6A). The average center of each slice was found for both sides and the distance from each centroid to that average was calculated. No significant differences were found in average-to-slice differences on the healthy side, likely due to large variability in the health side distances. Although the centroid location of Slice B varied substantially on the healthy side, there were no statistically significant difference between programs when taking the nerve as a whole. If this side were to be part of the clinical intervention, the individual results would call for closer examination of the nerve path. In contrast, a statistically significant difference was seen in two comparisons (P2&P4 and P1&P4) on the tumor side despite each slice showing differences of less than 2.0 mm (Fig. 6B).

While some statistically significant differences were found for local, linear measurements of tumors, they did not meet the threshold for clinical relevance based on the criteria for this anatomic ROI and case study. Specifically, the variance in tumor morphology and margins were below the expected clinical margin to be taken when excising the tumor and the location of the nerve did not change in a way that would modify a likely surgical plan. This emphasizes the importance of understanding the clinical relevance of measurements and not solely relying on statistical measures.

## Discussion

This study summarized segmentation and refining algorithm features, identified several critical points in data workflows that can introduce variation, and compared methods to measure variation between them when applied to the same data set. No single measurement was the best method for quantifying variation, so it was essential to have both global and local analyses of geometric differences. The intersection volumes and heatmaps were excellent tools to quickly identify the location and size of 3D differences between models. Local linear measurements of the affected nerve provided additional details that were useful in context. Several additional metrics were tried, but the combination of heatmap, volume, and disagreement metrics provided the most useful information for understanding variability introduced from algorithmic differences in the segmentation / refinement process.

Identifying and quantifying sources of variation is especially important when ground truth measurements cannot be made, as is the case with a patient’s internal anatomy. While cadaveric specimens or polymer models can provide independently verifiable measurements, they often do not fully replicate the living system. Therefore, users need to be able to assess the relative effects of different steps in their workflows to validate that it achieves the clinical needs of the model, while calling out areas where inherent variability will necessitate additional scrutiny. Combining available metrics provided enough local and global data to more holistically understand the variability introduced by different software programs and to identify potentially clinically relevant differences in model output. This study focused particularly on the effects of different software algorithms across programs. In addition to comparing algorithms, the same metrics can be applied to use of a single program with different settings to determine the effect of those changes (as done here with different levels of smoothing) or to determine the consistency between different users performing established procedures (not done in this study).

As outlined in the background, segmentation, smoothing, and decimation algorithms each have specific effects on the final output. It is critical that software programs preserve anatomical accuracy during the conversion process when these models are used for clinical decisions. Many software packages provide documentation of validation results and activities performed by the developers to verify their accuracy, including but not limited to receiving FDA clearance which reviews this factor. However, if these software packages are used off label for different, untested anatomy, it is incumbent that the user verify that the software provides a quality result. Products without FDA clearance may complete in-house or third-party testing to verify that they are accurate for specific use cases. In all cases, it is in keeping with both Good Laboratory Practices and Good Manufacturing Practices for each user or institution to test and validate any workflow they use.

Overall, it remains difficult to quantify variations in geometry created with different segmentation and smoothing techniques. Each anatomic ROI also has its own complexities and clinical requirements with regards to accuracy. To assess global differences between models, the volume and disagreement metrics provided quick and very useful overall measures of the match between different processes. In addition, the intersection models could be used to calculate a mismatch volume, surface area, and centroid. These could be particularly useful to monitor inter and intra user variability by setting a benchmark volume and disagreement metric for a standard scan, with users required to meet or beat that benchmark.

It is important to note that each metric has challenges as well. Volumes can only be calculated on closed surfaces, so some regions require additional manual intervention or automated repair before this metric can be calculated. We expected that the surface area metric would be more sensitive than the volume metric due to the irregular shapes in this ROI, however it was too sensitive to be useful. Small differences in the mesh, for example a sharp point or a jagged cluster of triangles, greatly affected the surface area with minimal real effect. In smooth and regular regions, surface area might be a suitable metric with appropriately high sensitivity. It is important to remember that even if a surface or volume metric shows statistically significant differences, those differences are not always clinically relevant and must be assessed according to the clinical needs determined beforehand for that ROI.

The heatmap view was one of the most robust, built-in tools to visualize differences between two models. Many mesh refinement programs include a heatmap generating function. Unlike other tools, they create a visual comparison of the entirety of two surface meshes. However, it is only useful in determining differences in model generation workflows for a given patient model and is not capable of determining the absolute accuracy of the surface mesh compared to the patient scan. Even so, heatmaps are still extremely useful in measuring process quality and repeatability, validating workflows, or assessing whether a new type of clinical case is within the capabilities of the existing protocols. Of note, heatmaps and the disagreement and intersection metrics require that both models be placed exactly in the same location. This is trivial when using a single scan but can be challenging when registering multiple imaging modalities. Model registration can also sometimes be done using built-in functions after surface meshes are created. However, if they are not available slice centroids and linear measurements may be more robust and effective metrics. The three-dimensional centroid and inertial coordinate system of each mesh can be found as previously described [57, 58].

As expected, regions of high geometric variation occurred in areas of high curvature, in agreement with other studies, [59] most notably in the channel formed by the tumor impinging on the nerve (Fig. 4B). Variation also existed in places susceptible to partial volume averaging, where a feature size was similar to or smaller than a given voxel size. This was illustrated in this use case in regions where there was thin residual cortical bone bordering the tumor. Visual inspection of the DICOM images showed a blurred boundary between the tumor and bone, implying that while the tumor had not breached the bone, it could not be segmented using standard thresholding because of how few voxels the bone occupied in comparison to surrounding soft tissue. When altering segmentation and refinement to include thin walls, care must be taken to maintain the boundaries of other similar tissues in the same area. Automatically processing features like thin walls requires specific quality controls or visual checks to prevent these features from being accidentally removed. While there are many benefits to automated methods, default settings are not always sufficient to preserve small or unexpected features. In many cases, operators must interpret the anatomic regions and modify the segmentation masks manually.

Linear measurements were the easiest way to assess the difference between specific points of interest. Multiple anatomical landmarks were tested for ease of repeatability, leading to the selection of those used in this study. Fiduciary markers should be selected for both clinical relevance and repeatability. If an anatomic region has a constant dimension in a critical location for the clinical intervention, then point dimensions are an easy way to assess accuracy (e.g., diameter of a vessel or length from a cut-plane to a nerve). Linear measurements can also be compared to clinical measurements made on the source imaging study, which provides an excellent means of confirming certain key features are accurate between the imaging and the model. It was noteworthy that higher smoothing levels produced larger differences between the models, likely because of the difference in the ways each program controlled for mesh shrinkage.

### Limitations

This paper investigates the effects of the implementation of different segmentation and refinement algorithms on the 3D model outputs. To ensure that the effects of the algorithms were isolated, a single segmenter and single data set were used. This paper cannot compare the magnitude of the algorithmic variation with inter-user or intra-user variability, between different scanning protocols, or in different clinical applications. In clinical use, these variables are comingled and often inextricable. Further studies identifying and using these parameters in the appropriate clinical context will be important to determining the overall effect of these algorithmic variations. However, this paper provides a baseline for understanding the magnitude of algorithmic effects and a set of measurement tools that, when used together, can provide enough information to make decisions as to model quality and process capability.

### Risk mitigation

Many studies have focused on dry bone models as their gold standard, but it has been established through this and other studies [60] that partial volume effects related to voxel size are a major cause of segmentation variation when soft tissue segmentation is necessary. Multiple prior studies have investigated the variations in printed anatomical models [10, 32, 55, 59], however, dry bone, ground truth studies cannot always account for the variability in clinical image volumes. Once a system’s physical accuracy and limits are established through a ground truth study, the accuracy of a patient-matching process typically requires more real-world data.

**Fig. 7:**
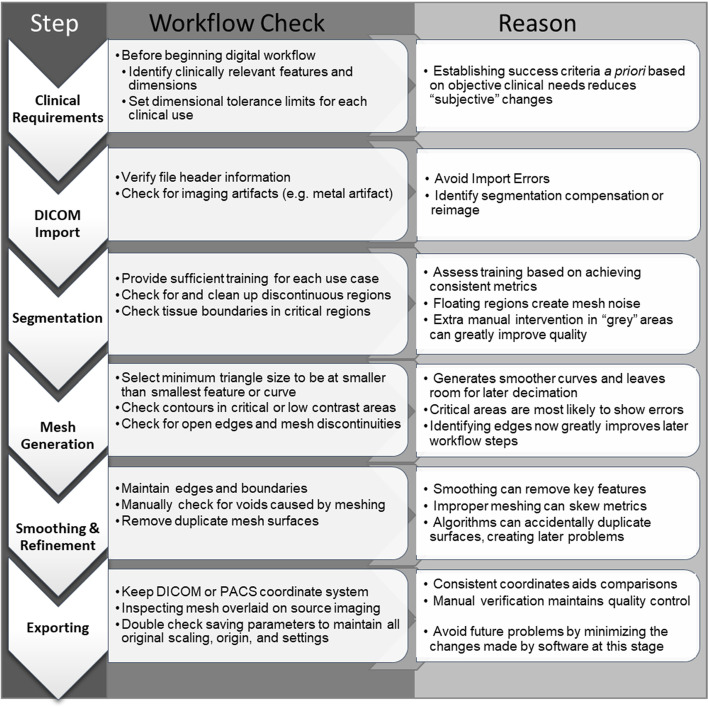
Check list to mitigate risk in digital workflow process. This is not an exhaustive list but provides basic checks for users to ensure that final STL files exhibit appropriate fidelity to original patient data

Minimizing the geometric variation during the DICOM to 3D model conversion process requires understanding of both the software workflow and the clinical needs. It can be thought of as mitigating risk to both the process and to the patient. This is best achieved by using staff trained well in both anatomy and segmentation processes to mitigate both software and inter-user variability. Models should always be assessed against patient data by appropriately trained engineers or clinicians. A checklist of workflow risk mitigations can be used and customized to fit each user’s needs (Fig. 7).

Software may be cleared by the FDA for specific applications or intended uses such as segmenting medical images to generate 3D digital models for clinical use. Some of the programs selected for this study have been FDA cleared for specific indications for use (IFU). In that case, the software has shown the FDA that it can create accurate 3D representations of the anatomy listed in its IFU. It also includes sufficient instructions to let trained users replicate those results. While it is not necessary for clinicians to use FDA-cleared software to design anatomic models, if they do not, they would then have to assume the burden of assessing and validating that their software is appropriate and accurate for their use cases. Validation of the software outputs may be done with the assistance of FDA guidance, but algorithm validation is generally left up to the specific vendor. For these reasons, there are benefits to both proprietary software where algorithms have gone through a thorough review process but whose inner workings are hidden, and open-source programs where users can see and control algorithm applications but may lack case-specific validation data. Regardless of the software choice, this study shows that an important aspect of producing models with minimal variability is the user’s understanding of the available parameters and how they function within the software of choice. Knowing when to apply and tweak specific parameters is essential to minimizing geometric variability between DICOM sources and final STL models. FDA clearance or third-party validation of software is dependent on the processes used and will not sufficiently mitigate risks unless users understand the algorithms the software applies and how certain parameters may impact the fidelity of the final model.

### Clinical relevance

The clinical relevance of geometric deviations in digital models is case dependent and should be assessed by qualified clinicians in reference to the specific procedure and anatomical region of interest. The digital conversion workflow should attempt to maintain the integrity of the original patient imaging data as much as possible. Tumor resection surgeries with larger surgical margins may not be as affected by small surface geometry errors as other surgeries with more stringent requirements. Clinical appropriateness criteria have been developed regarding which cases would benefit from patient-specific models [8].

To minimize the risk for error during digital model creation, it is essential to start with a patient scan of adequate resolution for segmentation. Minimizing voxel size while maintaining a sufficient signal to noise ratio is optimal for achieving high-quality segmentable data. Most diagnostic CT machines are capable of achieving slice thicknesses of less than 1 mm. Most patient-matched implants are currently accompanied by a specific scan protocol that is optimized for the anatomy around the implant location. Unique cases such as trauma, cancer, and congenital deformity that present some of the best potential for patient-matched technology do not yet have standardized requirements and should be evaluated based on clinical needs.

## Conclusions

Quantifying the variations in model design will be essential for patient-matched technology to reach maximum potential. This study provides comparisons of several metrics that can be used to validate methods of preparing patient-matched 3D prints. The software packages used here are representative of many that are available, all of which use similar mathematical foundations to identify regions of interest, then refine the regions to provide the best balance of accuracy and complexity. This provides very concordant results for regular and slowly varying smooth shapes. Most of the model disagreement then occurs during the refinement stages. Each program has features that refine models while reducing noise, preserving sharp curves, or maintaining the overall model volume. Any of these aspects may be desired or undesirable depending on the application. Once known, the implication of variation needs to be assessed by a qualified clinician per clinical application and often per case. Using whole-model metrics that can show the locations of variability (e.g. heatmaps and intersections) coupled with local slice-based measured (e.g. nerve slice centroids) balances the amount of measurement effort with information provided. For maintenance of a process, aggregate measures such as model volume and the disagreement metric were effective in identifying when one segmentation/refinement was sufficiently different from another to require additional checks. The algorithms and metrics described here can facilitation comparison of STL model consistency and accuracy regardless of workflow or program.

A basic understanding of the functionality of segmentation software is essential for ensuring patient safety as medical 3DP continues to expand. Methods used in this paper can help 3D printing facilities establish best practices for evaluating variation between segmentation methods and will allow users to develop optimized workflows—ideally accelerating the patient matched instrumentation implementation in industry and at point of care.

## Data Availability

DICOM data were used in a 2018 Radiological Society of North America training course and provided by Materialise NV. They are available upon request medical@materialise.be. Programs used and their specific versions are available from the authors upon request.

## Abbreviations

3DP: 3D Printing
AM: Additive Manufacturing
CT: Computed Tomography
DICOM: Digital Imaging and Communications in Medicine
FDA: US Food and Drug Administration
IFU: Indications for Use
MRI: Magnetic Resonance Imaging
ROI: Region of Interest
STL: Standard Tessellation Language
